# Malignancy Associated with Low-Risk HPV6 and HPV11: A Systematic Review and Implications for Cancer Prevention

**DOI:** 10.3390/cancers15164068

**Published:** 2023-08-11

**Authors:** Leandro Lima da Silva, Amanda Mara Teles, Joana M. O. Santos, Marcelo Souza de Andrade, Rui Medeiros, Ana I. Faustino-Rocha, Paula A. Oliveira, Ana Paula Azevedo dos Santos, Fernanda Ferreira Lopes, Geraldo Braz, Haissa O. Brito, Rui M. Gil da Costa

**Affiliations:** 1 Post-Graduate Program in Adult Health (PPGSAD), Federal University of Maranhão (UFMA), São Luís 65080-805, MA, Brazilana.azevedo@ufma.br (A.P.A.d.S.); haissa.brito@ufma.br (H.O.B.); 2 Post-Graduate Program in Animal Health, State University of Maranhão, São Luís 65099-110, MA, Brazil; 3 Molecular Oncology and Viral Pathology Group, Portuguese Institute of Oncology of Porto Research Center (CI-IPOP)/RISE@CI-IPOP (Health Research Network), Portuguese Institute of Oncology of Porto (IPO-Porto)/Porto Comprehensive Cancer Center (Porto.CCC), 4200-072 Porto, Portugal; 4 Centre for the Research and Technology of Agro-Environmental and Biological Sciences (CITAB), University of Trás-os-Montes and Alto Douro, 5000-801 Vila Real, Portugal; anafaustino.faustino@sapo.pt (A.I.F.-R.);; 5 Inov4Agro—Institute for Innovation, Capacity Building and Sustainability of Agri-Food Production, University of Trás-os-Montes and Alto Douro, 5000-801 Vila Real, Portugal; 6 Post-Graduate Program in Health Sciences, Federal University of Maranhão (UFMA), São Luís 65080-805, MA, Brazil; 7 Post-Graduate Program in Odontology, Federal University of Maranhão (UFMA), São Luís 65080-805, MA, Brazil; fernanda.ferreira@ufma.br; 8 Post-Graduate Program in Computing Sciences, Federal University of Maranhão (UFMA), São Luís 65080-805, MA, Brazil; geraldo.braz@ufma.br; 9 Laboratory for Process Engineering, Environment, Biotechnology and Energy (LEPABE), Faculty of Engineering, University of Porto, 4200-465 Porto, Portugal; 10 Associate Laboratory in Chemical Engineering (ALiCE), Faculty of Engineering, University of Porto, 4200-465 Porto, Portugal

**Keywords:** vaccine, squamous cell carcinoma, papillomavirus, retinoblastoma protein, low-risk HPV

## Abstract

**Simple Summary:**

Vaccination against human papillomavirus (HPV) helps prevent cancer caused by this virus. Determining which viral genotypes should be included is key for developing successful vaccination strategies. Low-risk genotypes, especially HPV6 and HPV11, are associated with benign warts. However, some studies also report their presence in cancers. We reviewed the scientific literature to estimate the proportion of cancers that bear single or dual HPV6/11 infections. HPV6 and HPV11 have been reported in up to 5.5% of penile and 87.5% of laryngeal cancers; however, they have not been reported in vulvar, vaginal or oral cancers. Next, we compared the HPV6/11 genomes with HPV16, the most common high-risk HPV genotype, and observed that the similarities mainly involved the *E7* gene, suggesting a limited ability to interfere with the differentiation of the host cells. These findings support the use of HPV vaccines that cover HPV6/11 not only for preventing genital warts but also for preventing specific types of cancers.

**Abstract:**

High-risk human papillomavirus (HPV) is etiologically related to cervical cancer, other anogenital cancers and oropharyngeal carcinomas. Low-risk HPV, especially HPV6 and HPV11, cause genital warts and laryngeal papillomas. However, the accumulating data suggests that HPV6 and HPV11 may cause malignant lesions at non-cervical anatomic sites. This review aims to estimate the proportions of single and dual HPV6/11 infections in multiple cancers reported in the last 10 years in the Cochrane, Embasa and PubMed databases. Secondly, the genomes of HPV6/11 were compared with the most common high-risk genotype, HPV16, to determine the similarities and differences. A total of 11 articles were selected, including between one and 334 HPV+ cancer patients. The frequencies of single or dual HPV6/11 infections ranged between 0–5.5% for penile and 0–87.5% for laryngeal cancers and were null for vulvar, vaginal and oral cancers. The genomic similarities between HPV6/11 and HPV16 mainly involved the *E7* gene, indicating a limited ability to block cell differentiation. The presence of single or dual HPV6/11 infections in variable proportions of penile and laryngeal cancers support the vaccination strategies that cover these genotypes, not only for preventing genital warts but also for cancer prevention. Other risk factors and co-carcinogens are likely to participate in epithelial carcinogenesis associated with low-risk HPV.

## 1. Introduction

Human papillomaviruses are host species-specific, double-stranded DNA viruses that exhibit a conserved icosahedral morphology, ranging between 50–55 nm in diameter with a molecular weight of 5 × 10^6^ Da [1,2]. Infection through tissue microdamage allows the virus to gain access to basal keratinocytes in the epidermis and keratinized mucosae [3,4]. The HPV genome contains a set of genes that are expressed early in the viral cycle upon cell entry, designated the “early” (E) genes, and two “late” (L) genes, L1 and L2, which are expressed later in the viral cycle and encode the structural capsid proteins as well as the regulatory regions [5]. Based on their nucleotide sequence of the L1 gene, HPVs are divided into types which are grouped in five genera (alpha, beta, gamma, mu and nu), where alpha is the main genus, comprising the HPV types associated with the development of cervical cancer known as high-risk (HR) HPVs (e.g., HPV16 and HPV18) and the types associated with genital warts, termed low-risk (LR) HPVs (e.g., HPV6 and HPV11) [6,7,8,9,10]. The HPV types are defined based on a 10% variation in their L1 nucleotide sequence and may be further subdivided into variants with different biological properties based on smaller differences [11,12,13,14]. The HPV types may also be divided according to their target epithelial site, i.e., cutaneous versus mucocutaneous [15]. Quadrivalent and nonavalent HPV vaccines are protective against HPV6 and HPV11 infections along with infections by HR-HPVs, while the bivalent vaccine only targets HPV16 and HPV18 [16]. Low-risk genital types are often responsible for benign lesions, such as condyloma acuminata, and may also cause low-grade cervical dysplasia. However, the risk of developing invasive cervical carcinoma is low [17]. HPV6 and HPV11 are most frequently found in genital warts [18] and are also involved in respiratory papillomas [19,20]. In contrast, HR-HPVs are able to establish persistent infections; interfere with cell proliferation, differentiation and survival; and are the etiologic agents of cervical cancer [5]. However, in contrast with cervical cancer, a limited number of studies have found single infections caused by LR-HPV, specifically HPV6 and HPV11, in small proportions of some non-cervical anogenital cancers, such as vulvar [21] and penile [22] cancers. Such observations suggest the hypothesis that these two HPV types may exert a more significant oncogenic effect on those specific anatomic sites than in the uterine cervix. If this is the case, the knowledge of the proportion of cancers potentially associated with HPV6/11 would help tailor vaccination strategies, especially in world regions where such cancers are more common. The present work adopted two complementary approaches to study this hypothesis. First, we performed a systematic review of the scientific literature from the last 10 years to determine the prevalence of single or dual HPV6 and HPV11 infections in the sites of HPV-associated cancers. Secondly, the genomic organization of these viruses was comparatively studied against HPV16, the most common high-risk HPV genotype, to identify meaningful similarities and differences. Finally, based on the results from both these studies, the factors that may contribute to a possible oncogenic role for HPV6 and HPV11 were discussed.

## 2. Materials and Methods

### 2.1. Systematic Review of HPV6 and HPV11 in Cancer

The systematic review was performed in accordance with the preferred reporting items for systematic reviews and meta-analyses (PRISMA) guidelines [23], according to the following parameters. Population: HNSCCs, anal, cervical, penile, vaginal and vulvar cancer patients. Intervention: the frequency of HPV6 and HPV11 single infections. The search strategy contemplated three standard databases on biomedicine: PubMed, Embase and Cochrane, accessed in March and April 2022. The keywords “cancer AND HPV6” or “cancer AND HPV11” were applied. A total of 541 articles were retrieved from PubMed, 695 articles from Embase and 29 articles from Cochrane. Duplicated records were excluded based on the article’s bibliographic reference. The following inclusion criteria were established concerning the type of study (case series and case–control studies in humans), tumor sample type (formalin-fixed paraffin-embedded and fresh tissue biopsies), tumor location (head and neck, uterine cervix, anorectum, penis, prepuce, vulva and vagina), histological diagnosis (squamous cell carcinoma), HPV detection methodology performed (PCR-based or sequencing techniques) and the type of agents identified (the frequency of single or dual HPV6 and HPV11 infections reported with or without a report of the infections by other LR-HPV types). The exclusion criteria were a lack of histological confirmation of cancer, a lack of identification of single/dual HPV6 or HPV11 infections, case reports, review articles and meta-analyses. The abstracts and, when necessary, the materials and methods were analyzed to apply the inclusion and exclusion criteria.

### 2.2. Comparative Genomic Analysis of HPV6, HPV11 and HPV16

The HPV6 and HPV11 genomes were compared with HPV16, the most commonly identified high-risk HPV in cancer, to identify the similarities and differences in the key genes involved in the cell transformation. The complete genomes of HPV16 (NC_001526.4) and HPV6 (NC_001355.1) were retrieved from the RefSeq database, available at NCBI. The RefSeq database was unavailable for HPV11. Therefore, its complete genome was retrieved from the GeneBank database, available at NCBI (MW404328.1). Then, the complete genomes of HPV16, HPV6 and HVP11 were uploaded to the Proksee/CGView Server online tool, which is a system for genome assembly, annotation and visualization [24]. In this tool, BLAST (blastn) was used to identify the regions of similarity between the genomic sequences. The amino acid sequences of the early proteins E6, E7, E5A and E5B from HPV16 and HPV6 were retrieved from the RefSeq database, available at NCBI, and the proteins from HPV11 were retrieved from the GenePept database, available at NCBI. The blastp tool from NCBI was used to evaluate the similarities between the early protein sequences.

## 3. Results

### 3.1. Systematic Review of HPV6 and HPV11 in Cancer

**Figure 1:**
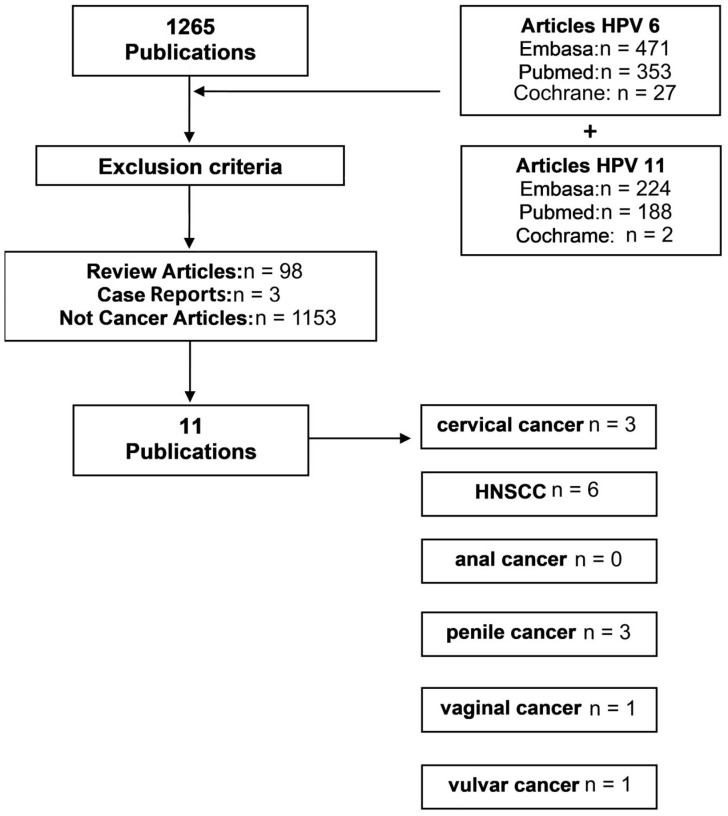
Systematic review of HPV6 and HPV11 in cancer and the resulting publications selected for analysis.

Most of the initially screened publications were excluded since they dealt with benign lesions instead of cancer, which was in line with the known role of HPV6 and HPV11 in warts. Overall, after applying the inclusion and exclusion criteria, 11 articles were selected for further analysis (Figure 1). A total of three articles were analyzed for cervical cancer [25,26,27], six for HNSCCs [28,29,30,31,32,33], none for anal cancer, three for penile cancer [28,34,35], one for vaginal cancer [28] and one for vulvar cancer [28]. The characteristics of all 11 publications that were selected for further analysis are summarized in Table 1.

The selected publications (Table 1) spanned the period between 2012 and 2021 and dealt with patient cohorts varying in size between eight and 1010 total patients. HPV detection was primarily performed using PCR-based methods, except for Aldersley et al. (2021) who used previously obtained whole exome data. The proportion of HPV-positive cases ranged between 1/85 [30] and 142/142 [25]. Seven studies used formalin-fixed paraffin-embedded (FFPE) material [28,29,30,31,32,34,35]; however, one used fresh biopsies [33] and another used tissue stored in RNAlater [26]. HPV genotyping was performed using a variety of commercial and custom methods. Three studies addressed the frequency of HPV6/11 infections in cervical cancer [25,26,27] (Table 2).

These studies generally identified an extremely low prevalence of single HPV6/11 infections or infections with HPV6/11 in the context of other LR-HPV in cervical cancer. A single case of HPV11 mono-infection was reported by [25], which also had the largest caseload of the three. Three studies addressed penile cancer [28,34,35]. Alemany et al. (2016) presented the largest caseload of the three and reported that 3.6% and 1.2% of HPV-positive cases carried HPV6 and HPV11 mono-infections, respectively. Barzon et al. (2014) reported a case showing HPV11 mono-infection and Magaña-León et al. (2015) reported none. In the larynx, the proportions of single infections varied between 0% and 75% cases for HPV6 and between 0% and 12.5% cases for HPV11. No studies observed single HPV6/11 infections in oral, vaginal or vulvar SCCs.

The HPV surrogate marker p16^INK4a^ was studied using immunohistochemistry in five articles. Two studies found that most penile cancers harboring HR-HPV were p16^INK4a^-positive [34,35]. However, the larger Alemany et al. [35] study found that only a small proportion of cancers with LR-HPV were p16^INK4a^-positive. Three studies described p16^INK4a^ immunostaining in laryngeal cancers [29,30,31] and reported a poor correlation with the presence of HPV DNA. Weiss et al. found two positive LR-HPV cases [31].

### 3.2. HPV6/11/16 Comparative Genomic Analysis

**Figure 2:**
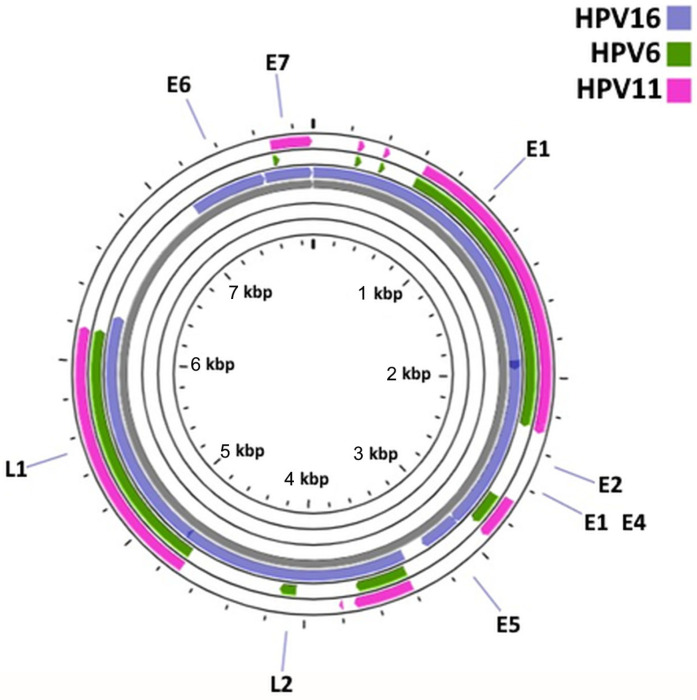
Comparative genomic organization of HPV6, HV11 and HPV16.

HPV16 had a circular dsDNA with a total of 7906 bp and eight coding sequences (Figure 2). When performing a blastn analysis at Proksee/CGView to compare both the HPV16/HPV6 and HPV16/HPV11 genomes, it was possible to observe that the majority of the similarities between the genomic sequences were located in the E1, E2, L2, L1 and E7 coding regions. No similarities between the genomic sequences at the E6 and E5 regions were found using these tools. The early proteins from HPV16 and HPV6/11 were then evaluated regarding the similarity of their amino acid sequence (Supplementary Table S1) using blastp. The identities, positives and expected values are presented in Table 3 and Table 4, where the identity describes the similarities of the sequences (the number of identical amino acids) and positives correspond to the number of amino acids that were either identical or had similar chemical properties.

According to the data obtained, the protein with highest similarity between HPV16 and HPV6 was E7, while E5 was the protein with lowest similarity. Similar results were obtained for HPV16 and HPV11.

## 4. Discussion

High-risk (HR)-HPVs, particularly HPV16, have been identified as the etiologic agents of multiple anogenital and oropharyngeal cancers, as shown by numerous observational and experimental studies [36,37,38,39,40,41]. LR-HPVs mostly cause benign lesions, such as condylomas, but have also been suggested to be involved in subsets of malignant non-cervical lesions [42,43]. The present work provided a systematic analysis of the data published in the last 10 years to determine the frequencies of HPV6 and HPV11 as single infections in anogenital and head and neck cancers. The 11 selected articles showed significant geographic diversity, including works from four continents, as well as a large international penile cancer study by Alemany et al. (2016). While some studies showed a low HPV-positive caseload for specific sites, such as the Weiss et al. (2015) and Magaña-Leon et al. (2015) reports, others were much larger and included dozens or hundreds of patients, such as the Taberna et al. (2016) or Alemany et al. (2016) articles. These heterogeneous results recommended caution when interpreting the findings from our systematic review. Smaller studies may highlight locally important phenomena, such as a higher HPV6/11 infection rate, while larger studies may dilute those observations and provide a more general picture. One study from Venezuela [33] reported data from multiple head and neck locations. However, it was impossible to ascribe specific HPV genotypes to each anatomical site. We chose to include this study because it provided data on the frequencies of LR-HPVs in head and neck SCCs in general. However, it could not replace the detailed reports ascribing HPV6 and HPV11 to more specific anatomic sites. We began by analyzing the studies focused on cervical cancer. Approx. 95% of women with cervical cancer were infected with one or more HR-HPV subtype, with HPV16 and 18 being the most common [44,45]. LR-HPV was associated with benign neoplasia and scientific data accumulated over decades does not support its involvement in cervical SCCs [46,47]. In line with such observations, our systematic review showed considerably low frequencies for HPV6 and HPV11 mono-infections in cervical cancer. Quadrivalent and nonavalent HPV vaccines conferred protection against these LR-HPV types and their associated lesions [16]. Advanced cervical cancer had dramatic consequences for patients due to cancer invasion and metastasis, but also due to psychological issues and paraneoplastic syndromes [48,49,50]. Even intraepithelial lesions were associated with significant morbidity, which could recur after surgical excision [51,52]. HPV-associated head and neck cancers were most frequently located in the oropharynx, where HPV16 was responsible for approximately 95% of the HPV-related cases [13,36,53]. The head and neck studies in our systematic review mostly reported data from the larynx, which likely reflected the known role of HPV6 and HPV11 in respiratory papillomas in this anatomic area [17]. HPV6 and HPV11 were the main causative agents of laryngeal papillomas, the most frequent benign tumors in the lower respiratory tract [17]. Respiratory lesions associated with the HPV11 type are suggested to be more aggressive compared to those associated with HPV6 [54]. Our systematic review showed that laryngeal SCCs carried HPV6 and HPV11 in varying proportions. While large studies from China and the USA showed frequencies ranging between 1% and 4%, a smaller German study showed much higher figures, with up to 75% of cases showing HPV11 mono-infections. It is likely that these widely varying figures reflected different geographical realities, but the results supported the involvement of HPV6 and HPV11 in a significant proportion of laryngeal SCCs. Other studies showed similar results [55]. The association between HPV6, HPV11 and this anatomic area may reflect local microenvironmental factors and an exposure to chemical carcinogens, as well as immunological impairment. These factors were not screened in this systematic review. Minimal data were available concerning other head and neck locations, with one study indicating null figures for oral SCCs [28] and the [33] study reporting figures for mixed locations. LR-HPV, such as HPV6 and HPV11, were associated with penile condylomas [56]. While it is possible that some penile condylomas may progress to SCCs [57], there is still insufficient data to support this hypothesis. Our systematic review showed that HPV6 and HPV11 infections were found in a significant proportion of penile SCCs, in agreement with the previous reports on penile cancer and penile intraepithelial neoplasia by multiple teams [58,59], including a meta-analysis by [22]. While the Magaña-Leon study with only eight cases did not identify any HPV6 orHPV11 single infections, larger studies such as those of Barzon et al. (2014) and especially Alemany et al. (2016), indicated that HPV6 and HPV11 mono-infections were found in approximately 5% of penile SCCs. Multiple LR-HPV infections were also found in 4% of other cases, according to Alemany et al. (2016). Taken together, these observations support the involvement of the LR-HPV types in a significant proportion of penile SCCs, suggesting that the penis and prepuce are anatomical sites with a particular susceptibility to carcinogenesis induced by these agents. Vaccines covering HPV6 and HPV11 may be more adequate for preventing penile neoplasia than bivalent vaccines targetting only HPV16 and HPV18. In the vulva and vagina, HPV6 and HPV11 were commonly associated with benign neoplasia, most often condylomas [47,60,61]. In our systematic review, a single study [28] addressed the frequency of HPV6 and HPV11 mono-infections in the vagina and vulva, limiting our ability to draw conclusions. This study suggested that a low frequency of infection caused by these LR-HPV types could be associated with vaginal SCCs. However, no cases of vulva SCCs with HPV6/11 were identified. These results were in agreement with the previous reports [62]. In our 10-year study period, no studies focused on anal cancer fulfilled the inclusion criteria, and we could not conclude the involvement of HPV6 and HPV11 mono-infections in this type of cancer. This was regrettable, as other studies identified the presence of a small proportion of anal SCCs associated with those LR-HPV types, especially in the context of immunosuppression induced by HIV [63,64,65,66].

It is possible that genomic similarities with high-risk HPV allow for HPV6 and HPV11 to interact with important cellular targets, conferring a limited carcinogenic potential. The HPV early proteins had regulatory functions and could be found in both high- and low-risk HPVs [67,68,69,70,71,72]. Among these, the E5, E6 and E7 oncoproteins were believed to be the main transforming proteins of HR-HPV [5]. While E5 may have a low transforming activity when expressed alone in a cell culture, it could play important roles in carcinogenesis induced by high-risk HPV [73,74]. The E6 protein was able to inactivate the p53 tumor suppressor protein and also perform p53-independent functions, thus playing a major role in the HPV-induced cell transformation [75,76]. The E7 protein played several major roles in carcinogenesis, especially by driving the degradation of the retinoblastoma protein (pRb) and thereby promoting cell proliferation [77,78]. It has been previously suggested that low-risk HPV types do not use their E6 and E7 gene products to drive extensive cell proliferation in the basal and parabasal cell layers, thereby drastically reducing their ability to induce cancer [79]. Indeed, our genomic analysis showed that the HPV6/11 and HPV16 genomes shared important differences concerning the E5, E6 and E7 oncogenes. However, they also exhibited some similarities. The E7 coding region was most conserved among all three viruses, while no similarities were found between the genomic sequences at the E6 and E5 regions. This suggests that the lower oncogenic potential of HPV6 and HPV11 compared with HPV16 was at least partly related to the differences on their E5 and E6 oncogenes. Conversely, the similarities observed in the E7 oncogene could help explain why HPV6 and HPV11 seemed to show some carcinogenic potential towards non-cervical tissues. Indeed, the HPV6 and HPV11 E7 proteins interacted with the pRb family member p130, inducing its proteasomal degradation. This mechanism could contribute to deregulating cell differentiation and proliferation in the suprabasal epithelial layers [80,81,82]. Classically, the accumulation of the p16INK4a protein was assessed immunohistochemically in squamous cell carcinomas as a surrogate marker for pRb downregulation to confirm viral activity [83]. Two of the studies included in this review [34,35] reported results for p16INK4a immunostaining in penile cancer, suggesting that only a minority of cases with LR-HPV were positive for this marker, which was in line with their limited ability to induce the degradation of pRb family proteins. Three studies of laryngeal cancer [29,30,31] reported that p16INK4a immunostaining had a poor correlation with the HPV DNA status in this type of cancer and, as observed for penile cancer, some LR-HPV-positive cases were p16INK4a-positive [29,31].

## 5. Conclusions

Overall, the present review combined and analyzed the data concerning the frequency of HPV6 and HPV11 mono-infections across multiple types of cancer. This analysis was limited by the small caseload of some studies and also by the absence of data concerning possible carcinogenic co-factors that could synergize with HPV6 and HPV11 to promote their tumorigenic potential. HPV6 and HPV11 mono-infections were mostly associated with SCCs of the larynx and penis. SCCs of the cervix, vagina, vulva and the head and neck (apart from the pharynx) showed the lowest frequencies of HPV6 and HPV11 mono-infections. It is plausible that factors such as immune suppression and specific changes to the local microbiome may contribute to the enhancement of viral persistence, while chemical agents may also act as co-carcinogens in the pharyngeal and penile mucosae. Establishing the etiologic role of these LR-HPVs in the penis and pharynx and the contributions of other co-factors will require additional studies and experimental demonstrations.

## Figures and Tables

**Table 1:** Characteristics of the 11 articles included in the HPV6 and HPV11 systematic review.

References	Type of Sample	Detection Method	Genotyping Method	Total Sample and (HPV + Sample)
Tao et al., 2017 [25]	Cervical scrappings	PCR/Luminex 200 (Tellgen, Shanghai, China)	PCR/Luminex 200 (Tellgen, China)	142 (142)
Das et al., 2013 [26]	Tissue biopsy in RNAlater (Qiagen, Hilden, Germany)	Nested PCR	Digene HPV Hybrid Capture II Test (Qiagen, Germany)	107 (105)
Aldersley et al., 2021 [27]	Whole exome data from previous publications	SureSelect Exon Capture (Agilent, Santa Clara, CA, USA) and HiSeq sequencing (Illumina, San Diego, CA, USA)	SureSelect Exon Capture (Agilent) and HiSeq sequencing (Illumina, USA)	72 (62)
Barzon et al., 2014 [34]	FFPE	PCR (Inno-LiPa, Tokyo, Japan)	Real-time PCR	59 (18)
Alemany et al., 2016 [35]	FFPE	SPF-10/DEIA/LIPA25 (Laboratory Biomedical Products, Rijswijk, The Netherlands)	LIPA25 (Laboratory Biomedical Products, The Netherlands) and Sanger sequencing	1010 (334)
Vietía et al., 2014 [33]	Fresh biopsies	PCR (Inno-LiPa, Japan)	PCR (Inno-LiPa, Japan)	71 (48)
Taberna et al., 2016 [29]	FFPE	PCR (Inno-LiPa, Japan)	Real-Time PCR	404 (54)
Lam et al., 2018 [30]	FFPE	Nested PCR	Sanger sequncing	85 (1)
Weiss et al., 2015 [31]	FFPE	Real-Time PCR GP5+/6+ and In Situ Hybridization	Real-Time PCR	8 (6)
Sun et al., 2012 [32]	FFPE	PCR for HPV 6/11 and HPV 16/18	PCR for HPV 6/11 and HPV 16/18	83 (42)
Magaña-León et al., 2015 [28]	FFPE	SPF-10/DEIA/LIPA25 (Laboratory Biomedical Products, The Netherlands)	PCR (Inno-LiPa, Japan)	35 (10)

**Table 2:** Prevalence of HPV6 and HPV11 single infections in different types of HPV-associated cancers.

Anatomic Location		HPV6 % (n/N)	HPV11 % (n/N)	Multiple Low-Risk% (n/N)	Geographical Location	References
Uterine cervix		0/142	1/142	1/142	China	Tao et al., 2017 [25]
		0/105	0/105	0/105	India	Das et al., 2013 [26]
		1.4% (1/62)	0/62	1/62	Republic of Korea/United States/France	Aldersley et al., 2021 [27]
Penis		0/18	5.5% (1/18)	1/18	Italy	Barzon et al., 2014 [34]
		3.6% (12/334)	1.2% (4/334)	4.8% (16/334)	25 Countries	Alemany et al., 2016 [35]
		0/8	0/8	0/8	Mexico	Magaña-León et al., 2015 [28]
Head and neck	Oral cavity	0/9	0/9	0/9	Mexico	Magaña-León et al., 2015 [28]
	Larynx	3.7% (2/54)	3.7% (2/54)	4/54	United States	Taberna et al., 2016 [29]
		1.2% (1/85)	0/85	1/85	China	Lam et al., 2018 [30]
		75.0% (6/8)	12.5% (1/8)	7/8	Germany	Weiss et al., 2015 [31]
		Not tested	Not tested	9.6% (8/42)	China	Sun et al., 2012 [32]
		0/9	0/9	0/9	Mexico	Magaña-León et al., 2015 [28]
	Mixed locations	12.5% (6/48)	0% (0/48)	16.67% (8/48)	Venezuela	Vietía et al., 2014 [33]
Vagina		0/7	0/7	1/7 *	Mexico	Magaña-León et al., 2015 [28]
Vulva		0/1	0/1	0/1	Mexico	Magaña-León et al., 2015 [28]

* One single HPV54 infection among seven vaginal SCC cases in the Magaña-Leon et al. (2015) study, which included 35 SCCs from multiple locations.

**Table 3:** A comparative analysis of the HPV6 and HPV16 E6, E7 and E5 oncoproteins.

		HPV6			
		E6 (NP_040296.1)	E7 (NP_040297.1)	E5A (NP_040301.1)	E5B (NP_040302.1)
HPV16	E6 (NP_041325.1)	Identities: 39%; Positives: 60%; Expect: 2 × 10^−41^			
	E7 (NP_041326.1)		Identities: 57%; Positives: 69%; Expect: 3 × 10^−34^		
	E5 (NP_041330.2)			Identities: 24%; Positives: 58%; Expect: 0.018	No significant similarity found.

**Table 4:** A comparative analysis of the HPV11 and HPV16 E6, E7 and E5 oncoproteins.

		HPV11			
		E6 (QXM18822.1)	E7 (QXM18823.1)	E5A (QXM18827.1)	E5B (QXM18828.1)
HPV16	E6 (NP_041325.1)	Identities: 37%; Positives: 61%; Expect: 2 × 10^−40^			
	E7 (NP_041326.1)		Identities: 55%; Positives: 70%; Expect: 1 × 10^−33^		
	E5 (NP_041330.2)			No significant similarity found.	No significant similarity found.

## Data Availability

The data produced in this study are available in this article and in Supplementary Table S1.

## Supplementary Materials

The following supporting information can be downloaded at: https://www.mdpi.com/article/10.3390/cancers15164068/s1, Table S1: HPV6, HPV11 and HPV16 E5, E6 and E7 protein sequences.
