# Supplementary material for: Malignancy Associated with Low-Risk HPV6 and HPV11: A Systematic Review and Implications for Cancer Prevention
Source: Cancers (Basel). 2023 Aug 11;15(16):4068. doi: 10.3390/cancers15164068 (PMC10452364; doi:10.3390/cancers15164068)
Supplement: Supplementary file 1 [file cancers-15-04068-s001.zip › cancers-2509817-supplementary.pdf]

Supplementary Table S1. HPV6, HPV11 and HPV16 E5, E6 and E7 protein sequences.

Gene	Protein sequence	HPV type	Reference
HPV16-E6	MHQKRTAMFQDPQERPRKLPQLCTELQTTIHDIILECVYCKQQLLRREVYD FAFRDLCIVYRDGNPYAVCDKCLKFYSKISEYRHYCYSLYGTTLEQQYNKPL CDLLIRCINCQKPLCPEEKQRHLDKKQRFHNIRGRWTGRCMSCCRSSRTRR ETQL	16	NP_041325.1
HPV16-E7	MHGDTPTLHEYMLDLQPETTDLYCYEQLNDSSEEEDEIDGPAGQAEPDRA HYNIVTFCCKCDSTLRLCVQSTHVDIRTLEDLLMGTLGIVCPICSQKP	16	NP_041326.1
HPV16-E5	MTNLDTASTTLLACFLLCFCVLLCVCLLIRPLLLSVSTYTSLIILVLLLWITAAS AFRCFIVYIIFVYIPLFLIHTHARFLIT	16	NP_041330.2
HPV18*-E6	MARFEDPTRRPYKLPDLCTELNTSLQDIEITCVYCKTVLELTEVFEFAFKDLF VVYRDSIPHAACHKCIDFYSRIRELRHYSDSVYGDTLEKLTNTGLYNLLIRCL RCQKPLNPAEKLRHLNEKRRFHNIAGHYRGQCHSCCNRARQERLQRRRET QV	18	NP_040310.1
HPV18*-E7	MHGPKATLQDIVLHLEPQNEIPVDLLCHEQLSDSEENDEIDGVNHQHLPAR RAEPQRHTMLCMCCKCEAIELVVESSADDLRAFQQLFLNTLSFVCPWCASQ Q	18	NP_040311.1
HPV18*-E5	MLSLIFLFCFCVCMYVCCHVPLLPSVCMCAYAWVLVFVYIVVITSPATAFTV YVFCFLLPMLLLHIHAILSLQ	18	NP_040315.1
HPV6*-E6	MESANASTSATTIDQLCKTFNLSMHTLQINCVFCKNALTTAEIYSYAYKHLK VLFRGGYPYAACACCLEFHGKINQYRHFDYAGYATTVEEETKQDILDVLIR CYLCHKPLCEVEKVKHILTKARFIKLNCTWKGRCLHCWTTCMEDMLP	6	NP_040296.1
HPV6*-E7	MHGRHVTLKDIVLDLQPPDPVGLHCYEQLVDSSEDEVDEVDGQDSQPLKQ HFQIVTCCCGCDSNVRLVVQCTETDIREVQQLLLGTLNIVCPICAPKT	6	NP_040297.1
HPV6*-E5	MMLTCQFNDGDTWLGLWLLCAFIVGMLGLLLMHYRAVQGDKHTKCKKC NKHNCNDDYVTMHYTTDGDYIYMN	6	NP_040302.1
HPV11*-E6	MESKDASTSATSIDQLCKTFNLSLHTLQIQCVFCRNALTTAEIYAYAYKNLK VVWRDNFPFAACACCLELQGKINQYRHFNYAAYAPTVEEETNEDILKVLIR CYLCHKPLCEIEKLKHILGKARFIKLNNQWKGRCLHCWTTCMEDLLP	11	QXM18822.1
HPV11*-E7	MHGRLVTLKDIVLDLQPPDPVGLHCYEQLEDSSEDEVDKVDKQDSQPLTQ HYQILTCCCGCDSNVRLVVECTDGDIRQLQDLLLGTLNIVCPICAPKP	11	QXM18823.1
HPV11*-E5A	MEVVPVQIAAATTTTLILPVVIAFAVCFLSIVLIILISDFLVYTSVLVLTLLLYLL LWLLLTTPLQFFLLTLCVCYFPAFYIHIYIVQTQQ	11	QXM18827.1
HPV11*-E5B	MVMLTCHLNDGDTWLFLWLFTAFVVAVLGLLLLHYRAVHGTEKTKCAKC KSNRNTTVDYVYMSHGDNGDYVYMN	11	QXM18828.1

HPV18*: Alphapapillomavirus 7: human papillomavirus 18 is a serotype of Alphapapillomavirus 10. HPV6*: Alphapapillomavirus 10: human papillomavirus 6 and 11 are serotypes of Alphapapillomavirus 10. HPV11*: Alphapapillomavirus 10: human papillomavirus 6 and 11 are serotypes of Alphapapillomavirus 10
